# Improving VTE Risk Assessments in Psychiatric Hospitals

**DOI:** 10.1192/bjo.2026.11391

**Published:** 2026-06-30

**Authors:** Aangelo Gurusinghe, Alan Smith

**Affiliations:** CNWL, London, United Kingdom

## Abstract

**Aims:**

Psychiatric inpatients face elevated risk of venous thrombo-embolisms due tosedation, antipsychotic use and reduced mobility. Following a serious incident involving a patient death from VTE complications, we aimed to increase VTE risk assessment completion within 14 hours of admission (KPI standard) from 74.7% to over 90% at St Charles Hospital and CNWL NHS Foundation Trust by November 2025.

**Methods:**

We conducted three PDSA cycles between November 2024 and November 2025, analysing 975 admissions across 8 psychiatric inpatient wards. Baseline compliance was 74.7% with 7.4% of patients never assessed.

Root cause analysis using fishbone diagrams identified gaps in staff awareness, competing clinical priorities and absence of electronic system prompts.

Cycle 1: We targeted education through training sessions reaching 80% of staff, resident doctor forums and targeting resident doctor inductions.

Cycle 2: We corrected the Tableau dashboard (data analytics platform) and started weekly automated emails for underperforming wards. With the aim of increasing data visibility.

Cycle 3: Implemented a mandatory soft-stop prompt in the SystmOne electronic patient record–which is triggered after prescribing. This was deployed as a regional trust-wide quality improvement intervention which affected all the inpatient beds at the CNWL trust (excluding CAMHS).

**Results:**

Education and awareness failed. Compliance fell 2.2 percentage points to 72.5%.

The data analytics dashboard visibility achieved partial improvement, raising compliance by 5.1 percentage points to 77.6%.

The semi-mandatory electronic prompt delivered the greatest impact, improving compliance by 9.6 percentage points to 87.2%.

Median time to assessment fell by 42%, from 1.30 hours to 0.76 hours.

The mean time fell from 58.1 to 9.1 hours; this indicates that there are far fewer extreme outliers who never receive VTE assessments.

Out-of-time assessments fell from 17.9% to 7.8%.

Overall compliance (comparing baseline to post cycle 3) rose from 74.7% to 87.2%.

The trust-wide implementation ensured consistent standards and an improvement in KPI across all inpatient wards.

**Conclusion:**

The system level change delivered better results than targeting human factors. Making VTE assessment semi-mandatory through an electronic prompt embedded into the clinical workflow and proved to be the best intervention. In addition the placement in the clinical workflow was designed to avoid prompt fatigue on the electronic systems and to ensure emergency prescribing was not interrupted.

This approach may benefit other mental health trusts seeking to improve VTE compliance. Further specific guidance on implementation can be provided for SystmOne orientated trusts.

